# Clinical relevance of glomerular IgM deposition in patients with lupus nephritis

**DOI:** 10.1186/s12865-021-00467-z

**Published:** 2021-12-07

**Authors:** Fengmei Wang, Jirong Yu, Lei Zhang, Yan Zhang, Jie Zhang, Bicheng Liu, Xiaowei Yang

**Affiliations:** 1 Institute of Nephrology, Zhong Da Hospital, Southeast University School of Medicine, Nanjing, China; 2 Department of Nephrology, The Fifth Affiliated Hospital of Xinjiang Medical University, Urumqi, 830000 Xinjiang China; 3 Department of Public Health, Aarhus University, Aarhus, Denmark; 4 Department of Nephrology, Provincial Hospital Affiliated to Shandong University, Jinan, 250021 Shandong China; 5 Department of Nephrology, Provincial Hospital Affiliated to Shandong First Medical University, Jinan, 250021 Shandong China

**Keywords:** IgM, Lupus nephritis, Complement factor H, Complement activation

## Abstract

**Background:**

The aim of the study was to investigate the clinical relevance of IgM deposition in patients with lupus nephritis (LN) in a large cohort.

**Results:**

217 patients with renal biopsy-proven active LN were enrolled. The associations between glomerular IgM deposition and clinicopathological parameters were further analyzed. IgM deposition was positively correlated with glomerular C1q and C3 deposition moderately (r = 0.436, *P* < 0.001; r = 0.408, *P* < 0.001, respectively), and inversely correlated with plasma levels of C3 and CFH mildly (r =  − 0.138, *P* = 0.043; r =  − 0.147, *P* = 0.037, respectively). By multivariate analysis, we found that glomerular IgM deposition independently contributed to glomerular C3 deposition in patients with LN (OR = 2.002, 95% CI 1.295–3.094, *P* = 0.002). In addition, we also found that patients with IgM 0–2+ had similar plasma CFH levels, but in patients with IgM3+–4+, plasma CFH levels were significantly lower (300.4 ± 155.8 μg/mL vs. 429.9 ± 187.5 μg/mL, *P* < 0.001). Furthermore, patients with high density of glomerular IgM and low levels of CFH had heavier proteinuria, higher serum creatinine and lower plasma C3 levels (5.7 ± 3.1 g/d vs. 4.7 ± 3.5 g/d, *P* = 0.037; 150.1 ± 121.0 μmol/L vs. 105.6 ± 97.1 μmol/L, *P* = 0.005; 0.3 ± 0.2 μg/L vs. 0.4 ± 0.2 μg/L, *P* = 0.04, respectively), comparing with those with low density of glomerular IgM and low levels of CFH.

**Conclusions:**

Our results suggested the involvement of glomerular deposited IgM in complement activation and renal injury in LN.

## Background

Lupus nephritis (LN) is the most common complication of systemic lupus erythematosus (SLE). The pathogenesis of LN involves initiation by immune complexes, activation of the immune system in the kidney, and the responses of renal parenchymal cells to these insults. Although LN is characterized by a “full-house” pattern of immune deposits, it is mostly suggested to be initiated by the glomerular deposition of nephritogenic IgG type autoantibodies at present [1–4].

Glomerular IgM deposition occurs in a wide range of glomerular diseases. It was previously considered to be passively trapped in areas of glomerulosclerosis. However, recent studies found that IgM specifically bound insulted glomeruli and exacerbated renal injury. In mice deficient in the complement factor H (CFH), a model of non-sclerotic and nonimmune-complex glomerular disease, IgM was identified as binding to glomerular epitopes and contributing to the progression of glomerular damage [5]. In another animal model of adriamycin-induced focal segmental glomerulosclerosis (FSGS), IgM deposition activated the complement system and mediated glomerular injury [6]. In the subsequent clinical studies, IgM deposition independently associated with worse renal outcomes in patients with various glomerular diseases, including FSGS, IgA nephropathy (IgAN), and diabetic nephropathy (DN) [7–9].

Since natural antibody IgM is suggested to bind to endogenous neoepitopes that are exposed after injury and exacerbates damage [10–13], whether it is involved in the pathogenesis of LN presented with various types of kidney injury deserves to be investigated. In our previous study, we found that plasma CFH levels in patients with LN at active phase were significantly lower than those in non-renal SLE patients or those in normal controls, and plasma CFH levels were negatively associated with SLEDAI scores and positively associated with serum C3 levels. In the remission phase of LN, plasma CFH returned to the normal levels [14]. Whether the decreased CFH level in active LN leads to extra glomerular IgM deposition just as it did in *CFH*^−/−^ mice is also an interesting question.

In this study, we firstly investigated the clinical relevance of renal IgM deposition and the relationship of plasma CFH levels with glomerular IgM deposition in a large Chinese LN cohort.

## Results

### Baseline data of patients with LN

In total, 217 consecutive patients with renal biopsy-proven active LN were enrolled. General clinical and renal histopathological profiles of the patients at renal biopsy are listed in Tables 1 and 2. The age of the patients were 32.7 ± 11.9 years old. The female-to-male ratio was 5.2:1. At the time of renal biopsy, all the patients recieved oral predisone or intravenous methyprednisonlone. 23 (10.6%) patients received mycophenolate mofetil combined with glucocorticoid, 12 (5.5%) patients received tarcrolimus combined with glucocorticoid, the other 182 patients received no immunosuppressants.

**Table 1:** General clinical profiles of patients with lupus nephritis at renal biopsy

Characteristic	Value
Number of patients	217
Age (mean ± s.d.) (years)	32.7 ± 11.9
Gender (female/male)	182/35
The time between presentation of lupus nephritis and biopsy (median, range) (months)	23 (0–214)
Duration of follow-up (median, range) (months)	49 (6–240)
SLEDAI (mean ± s.d.)	17.5 ± 5.7
Number of fever (non-infectious) (%)	66 (30.4)
Number of eruption (%)	120 (55.3)
Number of photosensitivity (%)	50 (23)
Number of oral ulcer (%)	64 (29.5)
Number of arthralgia (%)	118 (54.4)
Number of neurological disorder (%)	12 (5.5)
Number of anemia (%)	143 (65.9)
Number of thrombocytopenia (%)	66 (30.4)
Number of leukocytopenia (%)	101 (46.5)
Number of hematuria (%)	166 (76.5)
Number of leukocyturia (non-infection) (%)	113 (52.1)
Number of acute renal failure (%)	40 (18.4)
Hemoglobin (mean ± s.d.) (g/L)	101.9 ± 25.8
Urine protein (mean ± s.d.) (g/24 h)	4.3 ± 3.2
Serum creatinine (median, IQR) (μmol/L)	84 (68–129)
C3 (mean ± s.d.) (g/L)	0.46 ± 0.23
C4 (mean ± s.d.) (g/L)	0.23 ± 0.17
Antinuclear antibody positive (%)	214 (98.6)
Anti-dsDNA antibodies positive (%)	152 (70.0)
Anti-Smith antibodies positive (%)	51 (23.5)
Anti-SSA antibodies positive (%)	101 (46.5)
Anti-SSB antibodies positive (%)	24 (11.1)
Anti-C1q antibodies positive (%)	90 (41.5)

*s.d.* standard deviation, *SLEDAI* Systemic Lupus Erythematosus Disease Activity Index, *IQR* interquartile range, *dsDNA* double-stranded DNA, *SSA* Sjogren’s syndrome A antigen, *SSB* Sjogren’s syndrome B antigen

**Table 2:** Renal histopathological profiles of patients with lupus nephritis at renal biopsy

Characteristics	Value
Activity indices score (median, range)	8 (0, 19)
Endocapillary hypercellularity (median, range)	3 (0, 3)
Cellular crescents (median, range)	0 (0, 6)
Karyorrhexis/fibrinoid necrosis (median, range)	0 (0, 6)
Subendothelial hyaline deposits (median, range)	1 (0, 3)
Interstitial inflammatory cell infiltration (median, range)	1 (0, 3)
Glomerular leukocyte infiltration (median, range)	1 (0, 12)
Chronicity indices score (median, range)	2 (0, 10)
Glomerular sclerosis (median, range)	0 (0, 3)
Fibrous crescents (median, range)	0 (0, 3)
Tubular atrophy (median, range)	1 (0, 3)
Interstitial fibrosis (median, range)	1 (0, 3)

According to the 2003 classification of LN, 7 patients were classified as Class II (3.2%), 37 as Class III (17.1%, including 12 as Class III + V), 126 as Class IV (58.1%, including 7 as Class IV + V), and 47 as Class V (21.7%). None were Classes I and VI in this study.

### Glomerular IgM deposition and correlations with clinicopathological parameters in patients with LN

Renal immunofluorescence profiles of the 217 patients at renal biopsy are listed in Table 3. Among the 217 patients, 33 (15.2%) patients had no IgM deposition in glomeruli, whereas 184 patients had IgM deposition in glomeruli, including 72 (33.2%) patients with IgM 1+, 81 (37.3%) patients with IgM 2+, 29 (13.4%) patients with IgM 3+, and 2 (0.9%) patients with IgM 4+.

**Table 3:** Renal immunofluoresence profiles of patients with lupus nephritis at renal biopsy

Characteristics	Value
IgG immunofluorescence intensity, n (%)	
0+	8 (3.7)
1+	45 (20.7)
2+	78 (35.9)
3+	78 (35.9)
4+	8 (3.7)
IgA immunofluorescence intensity, n (%)	
0+	24 (11.1)
1+	57 (26.3)
2+	88 (40.6)
3+	46 (21.2)
4+	2 (0.9)
IgM immunofluorescence intensity, n (%)	
0+	33 (15.2)
1+	72 (33.2)
2+	81 (37.3)
3+	29 (13.4)
4+	2 (0.9)
C3 immunofluorescence intensity, n (%)	
0+	4 (1.8)
1+	21 (9.7)
2+	75 (34.6)
3+	107 (49.3)
4+	10 (4.6)
C1q immunofluorescence intensity, n (%)	
0+	15 (6.5)
1+	52 (24.0)
2+	83 (38.2)
3+	66 (30.4)
4+	2 (0.9)

To explore the clinical implications of glomerular IgM deposition in LN, we analyzed the correlations between the intensity of IgM deposition and clinico-histological manifestations of patients at biopsy. The percentage of glomerular IgM 3+–4+ in patients with histological Class II, III (including III + V), IV (including IV + V) and V were 14.3%, 13.5%, 16.7% and 8.5%, respectively. Although the percentage of stronger IgM deposition seemed to be lower in Class V, but the difference did not reach statistical significance (8.5% in Class V vs. 15.9% in other Classes; *P* = 0.201). It was found that the intensity of IgM deposition was correlated with the intensity of IgG and IgA deposition in glomeruli (r = 0.293, *P* < 0.001; r = 0.456, *P* < 0.001). IgM deposition was positively correlated with glomerular C1q and C3 deposition moderately (r = 0.408, *P* < 0.001; r = 0.436, *P* < 0.001, respectively), and inversely correlated with plasma levels of C3 and CFH mildly (r =  − 0.138, *P* = 0.043; r =  − 0.147, *P* = 0.037, respectively), but no correlation with plasma C1q levels (r =  − 0.109, *P* = 0.115). Among the pathologic indices, glomerular IgM deposition was positively correlated with subendothelial hyaline deposits mildly (r = 0.136, *P* = 0.045) and inversely correlated with interstitial inflammatory cell infiltration (r =  − 0.153, *P* = 0.025). There was no correlation between IgM deposition and proteinuria levels, serum creatine levels or SLEDAI scores (detailed in Table 4).

**Table 4:** Correlations between clinicopathological data and glomerular IgM deposition in lupus nephritis

Parameters	r	*P* value
Clinical parameters		
Age (years)	− 0.154	0.024
SLEDAI	0.013	0.847
Hemoglobin (g/L)	0.008	0.907
24-h urine protein (g/24 h)	0.050	0.466
Serum creatinine (μmol/L)	− 0.039	0.572
Plasma C1q levels (mg/L)	− 0.109	0.115
Plasma C3 levels (g/L)	− 0.138	0.043
Plasma CFH levels (μg/mL)	− 0.147	0.037
Plasma soluble C5b-9 levels (ng/mL)	0.009	0.899
Pathological parameters		
Histological classes	− 0.010	0.882
AI	0.003	0.965
Endocapillary hypercellularity	0.050	0.461
Cellular crescents	− 0.064	0.345
Karyorrhexis/fibrinoid necrosis	0.024	0.720
Subendothelial hyaline deposits	0.136	0.045
Interstitial inflammatory cell infiltration	− 0.153	0.025
Glomerular leukocyte infiltration	− 0.005	0.943
CI	− 0.022	0.745
Glomerular sclerosis	− 0.014	0.843
Fibrous crescents	− 0.012	0.860
Tubular atrophy	− 0.033	0.626
Interstitial fibrosis	− 0.062	0.360
Glomerular IgG deposition	0.293	< 0.001
Glomerular IgA deposition	0.456	< 0.001
Glomerular C3 deposition	0.436	< 0.001
Glomerular C1q deposition	0.408	< 0.001

*CFH* complement factor H, *SLEDAI* Systemic Lupus Erythematosus Disease Activity Index, *AI* activity indices, *CI* chronicity indices

### Glomerular IgM deposition independently contributes to glomerular C3 deposition in patients with LN

Complement activation was one of the mechanisms contributing to renal injury initiated by immune complexes deposition. Since glomerular IgM deposition was not only positively correlated with C3 deposition, but also correlated with glomerular IgG and IgA deposition, multivariate analysis was used to determine whether IgM contributed to complement activation independently.

As shown in Table 3, in our cohort, almost all the patients had C3 deposition in glomeruli. Among them, 46.1% patients had relatively mild C3 deposition (0+–2+), and 53.9% patients had relatively strong C3 deposition (3+–4+). Then multivariate analysis of predictors for strong granular C3 staining was performed using the binary logistic regression model. We found that glomerular IgM deposition independently contributed to glomerular C3 deposition in patients with LN (OR = 2.002, 95% CI 1.295–3.094, *P* = 0.002) (shown in Table 5).

**Table 5:** Risk factors for glomerular C3 deposition in patients with lupus nephritis

Parameter	Univariate analysis		Multivariate analysis	
	OR (95% CI)	*P* value	OR (95%CI)	*P* value
Sex, men	0.557 (0.262–1.187)	0.130	–	–
Age, per 10 yr	0.870 (0.682–1.108)	0.259	–	–
Urinary protein (g/24 h)	1.050 (0.972–1.135)	0.216	–	–
Serum creatinine (μmol/L)	1.002 (0.999–1.004)	0.171	–	–
Plasma C1q levels (μg/mL)	0.993 (0.980–1.007)	0.328	–	–
Plasma CFH levels (μg/mL)	0.999 (0.997–1.000)	0.123	–	–
Plasma C3 levels (g/L)	0.163 (0.047–0.559)	0.004	0.243 (0.052–1.138)	0.072
Glomerular C1q deposition	2.890 (2.890–4.158)	< 0.001	1.940 (1.281–2.937)	0.002
Glomerular IgG deposition	2.238 (1.600–3.130)	< 0.001	1.908 (1.278–2.848)	0.002
Glomerular IgA deposition	2.339 (1.677–3.263)	< 0.001	1.360 (0.915–2.021)	0.129
Glomerular IgM deposition	2.993 (2.073–4.322)	< 0.001	2.002 (1.295–3.094)	0.002

*CFH* complement factor H, *OR* odd ratio, *CI* confidence interval, *–* not included in the multiple analysis

### Association of glomerular IgM deposition and circulating CFH levels in patients with LN

**Fig. 1:**
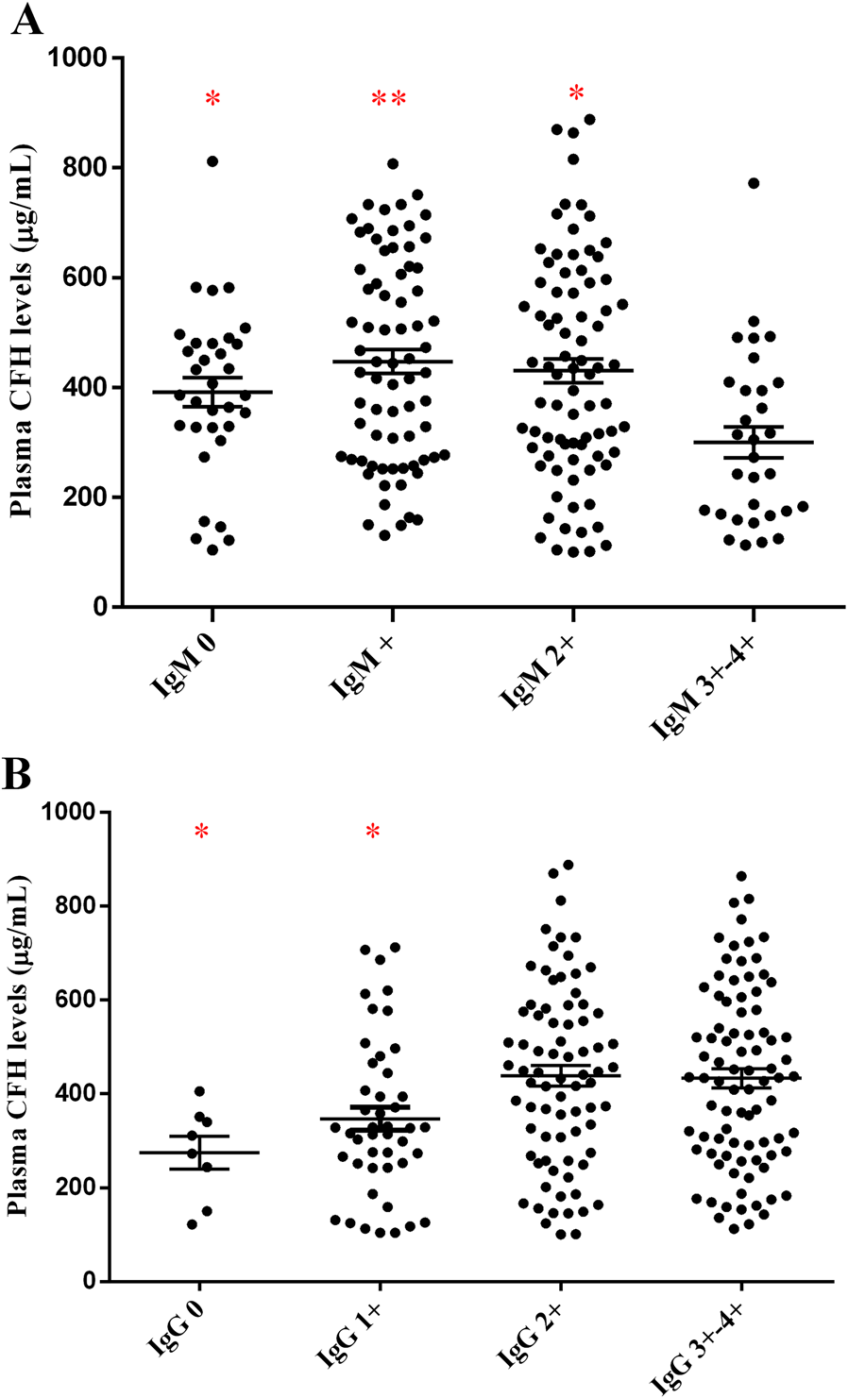
Plasma CFH levels in different groups. **A** Plasma CFH levels in different intensity of glomerular IgM deposition; **B** plasma CFH levels in different intensity of glomerular IgG deposition. *Note*: *P* value*: compared with the plasma CFH levels in lupus nephritis patients with glomerular IgM3+–4+ deposition or IgG 3+–4+. *: *P* < 0.05; **P < 0.001

In animal models, *CFH*^−/−^ leads to glomerular IgM deposition [5]. Interestingly, in our cohort, we also found negative correlation between the intensity of glomerular IgM deposition and plasma CFH levels, although the correlation coefficient was low. We further compared circulating CFH levels in patients with different glomerular IgM deposition intensity. Plasma CFH levels were significantly lower in patients with IgM 3+–4+ compared with those in patients with IgM 0+–2+ (300.4 ± 155.8 μg/mL vs. 429.9 ± 187.5 μg/mL, *P* < 0.001). While referring to IgG, it was found that plasma CFH levels were also associated with the intensity of glomerular IgG deposition, but with an opposite tendency compared with the deposition of IgM (Fig. 1).

### Association of clinicopathological parameters and the levels of plasma CFH and glomerular IgM deposition

According to our previous data, the median plasma CFH level in healthy controls was 542.8 μg/mL [14]. Then we divided our LN patients into 2 groups: Lower CFH level group (plasma CFH levels < 542.8 μg/mL) and higher CFH level group (plasma CFH levels ≥ 542.8 μg/mL). In this cohort, 165 patients had lower plasma CFH levels, and 52 patients had higher plasma CFH levels. In the higher CFH level group, 51 patients had relatively weaker glomerular IgM deposition (0–2+), and only 1 patient had glomerular IgM 3+.

In the lower CFH level group, 30 patients had glomerular IgM 3+–4+, while 135 patients had glomerular IgM 0–2+. We found that patients with stronger glomerular IgM deposition had significantly heavier proteinuria, higher serum creatinine and lower plasma C3 levels (5.7 ± 3.1 g/d vs. 4.6 ± 3.3 g/d, *P* = 0.026; 150.1 ± 121.0 μmol/L vs. 124.7 ± 140.6 μmol/L, *P* = 0.015; 0.30 ± 0.2 μg/L vs. 0.44 ± 0.22 μg/L, *P* = 0.023, respectively). There was no significant difference in other indices (detailed in Table 6).

**Table 6:** Comparisons of clinical, laboratory and pathological data in patients with lower plasma CFH level group

	Lower plasma CFH level group		
	IgM 3+–4+ N = 30	IgM 0–2+ N = 135	*P*
Clinical evaluation			
Gender (male %)	6 (20.0%)	17 (12.6%)	0.380
Age (mean ± s.d.) (years)	31.1 ± 10.5	33.3 ± 11.8	0.411
SLEDAI (mean ± s.d.)	17.2 ± 5.2	18.0 ± 5.8	0.408
Laboratory assessment			
Hemoglobin (mean ± s.d.) (g/L)	100.9 ± 26.5	99.6 ± 25.7	0.859
Urine protein (median; IQR) (g/24 h)	5.7 ± 3.1	4.6 ± 3.3	0.026
Scr (mean ± s.d.) (μmol/L)	150.1 ± 121.0	124.7 ± 140.6	0.015
Plasma C1q (mean ± s.d.) (μg/mL)	33.2 ± 18.8	33.2 ± 18.8	0.191
Plasma C3 levels (mean ± s.d.) (g/L)	0.30 ± 0.2	0.44 ± 0.22	0.023
Plasma C4 levels (mean ± s.d.) (g/L)	0.23 ± 0.17	0.21 ± 0.18	0.756
Plasma CFH levels (mean ± s.d.) (μg/mL)	294.6 ± 131.1	338.7 ± 124.3	0.052
Plasma soluble C5b-9 levels (mean ± s.d.) (ng/mL)	1225.9 ± 571.2	1271.4 ± 657.8	0.785
Pathological parameters			
AI (median; IQR)	8 (6, 10)	8 (4, 11)	0.695
Endocapillary hypercellularity	3 (2, 3)	3 (1, 3)	0.620
Cellular crescents	0 (0, 2)	0 (0, 2)	0.591
Karyorrhexis/fibrinoid necrosis	0 (0, 2)	0 (0, 2)	0.640
Subendothelial hyaline deposits	1 (1, 3)	1 (0, 2)	0.118
Interstitial inflammatory cell infiltration	1 (1, 1)	1 (1, 2)	0.856
Glomerular leukocyte infiltration	1 (1, 1)	1 (0, 1)	0.454
CI (median; IQR)	2.5 (2, 4)	2 (2, 4)	0.368
Glomerular sclerosis	0 (0, 1)	0 (0, 1)	0.970
Fibrous crescents	0 (0, 1)	0 (0, 0)	0.228
Tubular atrophy	1 (1, 1)	1 (1, 1)	0.481
Interstitial fibrosis	1 (1, 1)	1 (1, 1)	0.604

*CFH* complement factor H, *s.d.* standard deviation, *SLEDAI* Systemic Lupus Erythematosus Disease Activity Index, *IQR* interquartile range, *AI* activity indices, *CI* chronicity indices

### Association of glomerular IgM deposition and renal outcome in patients with LN

**Fig. 2:**
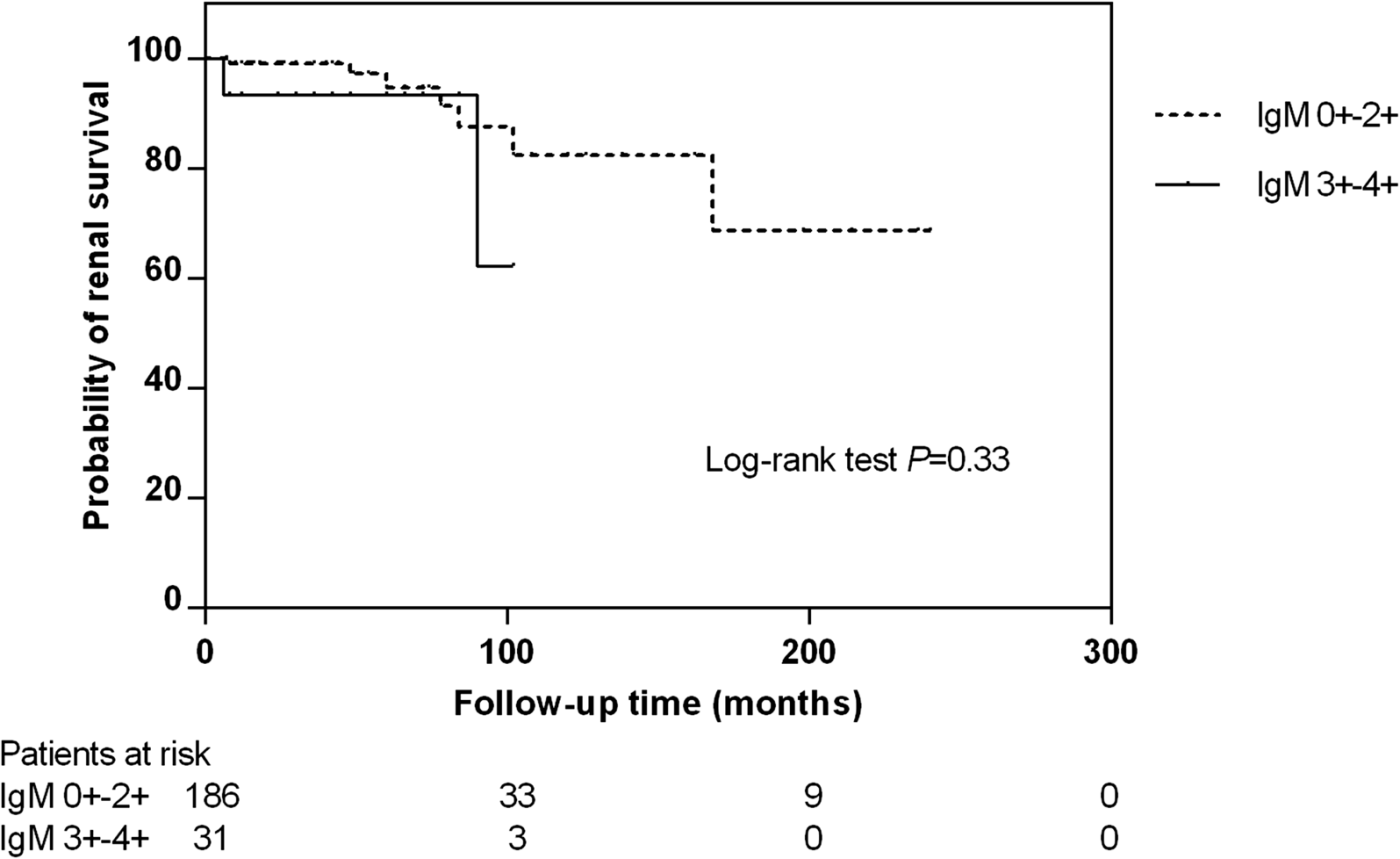
The correlation between intensity of glomerular IgM deposition and renal survival in patients with lupus nephritis

In our cohort, patients with LN were followed up for a mean duration of 57.3 ± 58.1 months after renal biopsy, and the median time between presentation of LN and renal biopsy were 23 months (0–214 months). The median duration of follow-up was 49 months, ranged from 6 to 240 months. We evaluated the association between glomerular IgM deposition and renal survival by Kaplan–Meier survival analysis. It was found that glomerular IgM deposition was not a risk factor for renal outcome in our patients with LN (*P* = 0.581, HR = 0.854, 95% CI 0.488–1.495). Patients were classified into two groups according to the intensity of glomerular IgM deposition: IgM 0+–2+ and IgM 3+–4+. However, there was still no significant difference in renal survival between the two groups (*P* = 0.33) (Fig. 2).

## Discussion

Glomerular IgM staining is commonly observed in the mesangial area and on the capillary wall of glomeruli in patients with LN, but its clinical significance has not been elucidated yet. In this large cohort of patients with LN, we firstly reported that glomerular IgM independently contributed to glomerular C3 deposition, and glomerular IgM deposition intensity was associated with plasma CFH levels.

The deposition of immune complexes and the subsequent complement activation are considered major mechanisms by which tissue injury occurs in LN [15–17]. Many studies have reported the pathogenic role of IgG type autoantibodies, such as anti-dsDNA antibodies, anti-C1q antibodies, anti-mCRP antibodies, in complement activation and renal injuries in LN [18–21]. Natural IgM antibody is generally regarded as an activator of complement classical pathway. Complement activation by IgM antibodies was essential for C3 deposition on apoptotic cells and their uptake by macrophages [22], indicating the involvement of IgM in the pathogenesis of SLE. In this study, we found that the intensity of glomerular deposited IgM was positively correlated with the intensity of glomerular deposited IgG and IgA, and moderately correlated with glomerular deposited C1q and C3. In the further multivariate analysis, glomerular deposited IgG and C1q contributed to glomerular C3 deposition, which supported that nephritogenic IgG autoantibodies mediated complement classical pathway was activated in renal tissue in LN. What’s more, IgM rather than IgA contributed to glomerular C3 deposition independently. Our findings firstly indicated that the abundant deposited IgM in glomeruli in LN was involved in the complement activation and pathogenicity.

Deletion of CFH results in uncontrolled complement alternative pathway activation and glomerular injury. *CFH*^−/−^ mice is an animal model of non-sclerotic and nonimmune-complex glomerular disease. CFH deficiency induced IgM deposition on endothelial cells and subendothelial areas. Panzer et al. crossed *CFH*^−/−^ mice with *μMT* mice (mice unable to produce IgM) and demonstrated that IgM contributed to the progression of glomerular damage induced by CFH deficiency [5]. In active LN, plasma CFH levels decreased to lower levels [14]. Interestingly, in this study we found that glomerular IgM deposition was inversely correlated with plasma levels of CFH, plasma CFH levels were significantly lower in patients with stronger IgM deposition (3+–4+) compared with those in patients with weaker IgM deposition (0+–2+), and stronger glomerular IgM deposition was more often observed in lower CFH group. Our results proposed that in patients with LN, CFH deficiency seemed to induce extra glomerular IgM deposition too. IgM within the glomerulus was considered as a downstream event occurring secondary to glomerular damage. Some clinical studies of patients with nephrotic syndrome demonstrated more severe manifestations in association with secondary glomerular IgM deposition [23–26]. However, it is clear that not all patients with glomerular disease develop IgM deposition. According to the result of animal model and this clinical study, the correlationship of secondary IgM deposition and plasma CFH levels was deserved to be explored in other glomerulonephritis.

LN is characterized by the “full-house” pattern of immune deposits, IgM deposition in LN was obviously not only induced secondarily. In our cohort, it was not found obvious correlations between IgM deposition and clinicopathological parameters. As discussed above, lower CFH levels might cause extra IgM deposition. We further compared clinicopathological parameters in patients with lower CFH levels, and found that patients with stronger glomerular IgM deposition had significantly heavier proteinuria, higher serum creatinine and lower plasma C3 levels in the analysis subgroup. Our results indicated that secondarily deposited IgM in LN might also demonstrate more severe manifestations.

On the basis of the above relevance of glomerular IgM deposition in patients with LN, it is important to evaluate the predictive value of glomerular IgM deposition for long-term renal outcome in LN. Recently, several studies showed that IgM deposition predicted renal outcome in patients with IgAN, FSGS and diabetic nephropathy [7, 9, 27, 28]. However, owing to the relatively good treatment response of LN and our relatively small follow-up population, we failed to draw any convincing conclusions regarding the predictive value of glomerular IgM deposition in our study.

Our study had several limitations. First, it was a retrospectively observational study, a cause-effect relationship could not be established. Second, this is the first report revealing clinical significance of IgM deposition in patients with LN, findings from this single-center study require validation from multicenter studies with larger cohorts. Third, a “full-house” pattern of immune deposits in LN caused complex pathophysiological process in complement activation, IgM deposition involved in kidney injury progression was not fully interpreted.

In conclusion, this study shows that glomerular IgM independently contributed to glomerular C3 deposition, and glomerular IgM deposition intensity was associated with plasma CFH levels. The findings indicate the involvement of glomerular IgM in complement activation and renal injury.

## Methods

Informed consent was obtained for blood sampling and renal biopsy from each patient. For participants under 16 years old, written informed consent was provided by a parent or guardian. In addition, all clinical test results were also obtained after patients gave their consent to use them for research purposes. The research was in compliance with the Declaration of Helsinki and approved by IEC for Clinical Research of Southeast University affiliated Zhongda Hospital (No. 2013-075).

### Patients

A total of 217 consecutive patients with renal biopsy–proven lupus nephritis diagnosed at Zhongda Hospital affiliated to Southeast University from 6 September 2013 to 2 March 2019 were enrolled in this study. The patients all fulfilled the 1997 ACR revised criteria for SLE [29].

### Clinical evaluations

The following clinical data were collected and analysed: gender, age, fever, anaemia, leucocytopenia, thrombocytopenia, haematuria, leukocyturia, 24-h proteinuria, serum creatinine and hemoglobulin. Clinical disease activity was assessed using the SLEDAI [30]. eGFR was calculated using a Scr-based equation adjusted for coefficients for age and gender by modified abbreviated MDRD equation based on data from Chinese CKD patients: eGFR (mL/min per 1.73m^2^) = 175 × [Scr (mg/dL)]^−1.234^ × age^−0.179^ × (0.79 if female) [31].

The criteria for clinical remission were the same as we reported previously [14]. The remission of lupus nephritis includes complete remission and partial remission. Complete remission was defined as a urinary protein excretion of < 0.3 g/day, with normal urinary sediments (red blood cell < 3/high power (HP), white blood cell < 5/HP), serum albumin and renal function. Partial remission was defined as the presence of any one of the following features: a decrease in serum creatinine to below 130 μmol/L for patients with a baseline serum creatinine of ≥ 130 μmol/L, but ≤ 260 μmol/L; a decrease in serum creatinine by > 50% for patients with a baseline serum creatinine of > 260 μmol/L; a decrease in urinary protein excretion by > 50%, and below 3.0 g/24 h, with a serum albumin of ≥ 30 g/L and stable renal function.

The patients were followed up in our outpatient clinic specified for LN. The renal endpoints were defined as end-stage renal disease (ESRD) or doubling of serum creatinine.

### Blood samples

Plasma and sera from patients with LN were obtained from peripheral blood at the time of renal biopsy. All the blood samples were stored at − 80 °C until use.

### Laboratory assessment

Serum ANAs, anti-dsDNA antibodies, anti-extractable nuclear antigen antibodies, including anti-Sm, anti-SSA, anti-SSB antibodies, were detected using immunodotting assays (EUROIMMUN, Lübeck, Germany; normal range). Plasma C3 was determined using a rate nephelometry assay (BeckmanCoulter, IMMAGE, USA; normal range > 0.85 g/L).

Anti-C1q IgG autoantibodies were detected using a previously published ELISA method [32]. The results were recorded as the net optical absorbance (average value of antigen wells minus average value of antigen-free wells) at 490 nm in an ELISA reader (BioRad 550, Japan) and expressed as percentage of the known positive sample. The cutoff value was set as the mean + 2 SD of healthy blood donors.

### Quantification of plasma levels of complement components

Plasma concentrations of human complement components were determined by enzyme-linked immunosorbent assay, including plasma C1q, plasma CFH and soluble C5b-9 (Quidel Corporation, San Diego, CA). The detection of plasma soluble C5b-9 was assayed in accordance with the manufacturer's instructions.

The method to detect plasma C1q was modified as previously described [32]. Rabbit anti-human C1q polyclonal antibodies (Dako, Denmark) were coated on to the microtiter plates (Nunc Immunoplate, Roskilde, Denmark) overnight at 4 °C. Then the wells were washed 3 times with 0.01 M phosphate-buffered saline (PBS) containing 0.1% Tween20 (PBST) and blocked with PBSTcontaining1% bovine serum albumin (BSA). After standards and serum samples added, horseradish peroxidase–conjugated goat anti-human C1q monoclonal antibody (Abcam, US) was added and incubated. The reaction was developed with tetramethylbenzi-dine (TMB) liquid substrate system and was stopped with 1 M H_2_SO_4_. The results were recorded as the net optical absorbance at 450 and 570 nm in an ELISA reader (Bio-Rad550, Japan).

The method of detecting plasma CFH was the same as previously described [14]. The CFH level of each sample was calculated using Curve Expert 1.3 (Hyams Development, http://www.curveexpert.net/). All assays were run in duplicate, and when standard errors were > 10%, samples were routinely re-analyzed. Serial concentrations of commercially available highly purified human factor H were used to develop a standard curve. The linear portion of the curve was subsequently used for the measurement of plasma factor H.

### Renal histopathology

The renal biopsy specimens were examined by light microscopy, direct immunofluorescence and electron microscopy. LN was re-classified according to the International Society of Nephrology/Renal Pathology Society (ISN/RPS) 2003 classification system [33]. All renal histopathological data of 217 patients with renal biopsy-proven lupus nephritis, were reviewed by two pathologists with 20-year experience. The pathologists classified and scored the biopsies separately, blinded to patients’ data and the scores of the other observer. Differences in scoring between the pathologists were resolved by rereviewing the biopsies and reaching a consensus.

### Intra- and inter-reader reliability

The two pathologists who undertook the analyses of the pathological data, were unaware of the patients’ details. Each evaluation was performed in triplicate and the mean of the values were reported. In addition, in each patient’s pathological data evaluation, two expert pathologists reported their results. The inter-rater reliability for immunofluorescence IgG and IgM scores was good, Cohen's kappa = 0.953 (*P* < 0.001), Cohen's kappa = 0.951 (*P* < 0.001), respectively. The intra-rater reliability for immunofluorescence IgG and IgM scores from one pathologist was excellent, ICC = 0.962 (95% CI 0.952–0.971), ICC = 0.980 (95% CI 0.974–0.985), respectively. And the intra-rater reliability for immunofluorescence IgG and IgM scores from the other pathologist was also good, ICC = 0.946 (95% CI 0.931–0.958), ICC = 0.965 (95% CI 0.955–0.973), respectively.

### Light microscopy examination

Renal biopsy specimens were fixed in 4.5% buffered formaldehyde for light microscopy. Consecutive serial 3 µm sections were used for histological staining. Stains employed included hematoxylins and eosin (H&E), periodic acid-Schiff, silver methenamine (Meth) and Masson’s trichrome.

### Immunofluorescence examination

The intensity of immunofluorescence for IgG, IgA, IgM, C3, C1q, fibrin, kappa and lambda deposits were semi-quantitatively graded from 0 to 4, respectively. Staining intensity was expressed as follows: 0, invisible; 1+, ambiguous under a low-power lens and apparent under a high-power lens; 2+, apparent under a low-power lens and clear under a high-power lens; 3+, clear under a low-power lens and bright under a high-power lens; and 4+, dazzling under a high-power lens.

### Electron microscopy

Renal biopsy specimens were fixed in 2.5% paraformaldehyde for electron microscopy. After embedded in epon, ultrathin sections were mounted on metal grids and stained with uranyl acetate before viewed in a transmission electron microscope (JEM-1230; JEOL,Tokyo, Japan).

### Statistical analysis

Statistical software SPSS 25.0 (SPSS, Chicago, IL, USA) was employed for all the statistical analysis. Quantitative data were expressed as mean ± s.d., median with interquartile range (IQR), median with range, or number (%). One-way analysis of variance was used for the same continuous data in different groups. Differences of quantitative variables between groups were assessed using the t test (for normally distributed data) or Mann–Whitney U test (for non-normally distributed data). Logistic regression analysis was carried out to predict glomerular C3 deposition. Survival analysis was performed using the log-rank test. Statistical significance was considered as *P* < 0.05.

## Data Availability

Further clinical data and images of this case are available from the corresponding author upon reasonable request.

## Abbreviations

LN: Lupus nephritis
SLE: Systemic lupus erythematosus
CFH: Complement factor H
FSGS: Focal segmental glomerulosclerosis
IgAN: IgA nephropathy
DN: Diabetic nephropathy
SLEDAI: Systemic Lupus Erythematosus Disease Activity Index
dsDNA: Double-stranded DNA
SSA: Sjogren’s syndrome A antigen
SSB: Sjogren’s syndrome B antigen
AI: Activity indices
CI: Chronicity indices
PBS: Phosphate-buffered saline
BSA: Bovine serum albumin
ISN/RPS: International Society of Nephrology/Renal Pathology Society
IQR: Interquartile range
OR: Odd ratio
CI: Confidence interval
AI: Activity indices
CI: Chronicity indices
